# Correlation between Phenotypic and In Silico Detection of Antimicrobial Resistance in *Salmonella enterica* in Canada Using Staramr

**DOI:** 10.3390/microorganisms10020292

**Published:** 2022-01-26

**Authors:** Amrita Bharat, Aaron Petkau, Brent P. Avery, Jessica C. Chen, Jason P. Folster, Carolee A. Carson, Ashley Kearney, Celine Nadon, Philip Mabon, Jeffrey Thiessen, David C. Alexander, Vanessa Allen, Sameh El Bailey, Sadjia Bekal, Greg J. German, David Haldane, Linda Hoang, Linda Chui, Jessica Minion, George Zahariadis, Gary Van Domselaar, Richard J. Reid-Smith, Michael R. Mulvey

**Affiliations:** 1 National Microbiology Laboratory, Public Health Agency of Canada, Winnipeg, MB R3E 3R2, Canada; aaron.petkau@phac-aspc.gc.ca (A.P.); ashley.kearney@phac-aspc.gc.ca (A.K.); celine.nadon@phac-aspc.gc.ca (C.N.); philip.mabon@phac-aspc.gc.ca (P.M.); jeffrey.thiessen@phac-aspc.gc.ca (J.T.); gary.vandomselaar@phac-aspc.gc.ca (G.V.D.); michael.mulvey@phac-aspc.gc.ca (M.R.M.); 2 Department of Medical Microbiology and Infectious Diseases, University of Manitoba, Winnipeg, MB R3E 0J9, Canada; 3 Centre for Food-Borne, Environmental and Zoonotic Infectious Diseases, Public Health Agency of Canada, Guelph, ON K1A 0K9, Canada; brent.avery@phac-aspc.gc.ca (B.P.A.); carolee.carson@canada.ca (C.A.C.); richard.reid-smith@phac-aspc.gc.ca (R.J.R.-S.); 4 United States Centers for Disease Control and Prevention, Atlanta, GA 30333, USA; lly3@cdc.gov (J.C.C.); gux8@cdc.gov (J.P.F.); 5 Cadham Provincial Laboratory, Winnipeg, MB R3E 3J7, Canada; david.alexander@gov.mb.ca; 6 Public Health Ontario Laboratories, Toronto, ON M5G 1M1, Canada; vanessa.allen@oahpp.ca; 7 Horizon Health Network, Saint John, NB E2L 4L2, Canada; sameh.elbailey@horizonnb.ca; 8 Laboratoire de Santé Publique du Québec, Sainte-Anne-de-Bellevue, QC H9X 3R5, Canada; sadjia.bekal@inspq.qc.ca; 9 Queen Elizabeth Hospital, Charlottetown, PE C1A 8T5, Canada; gjgerman@ihis.org; 10 Queen Elizabeth II Health Sciences Centre, Halifax, NS B3H 2Y9, Canada; david.haldane@cdha.nshealth.ca; 11 British Columbia Center for Disease Control, Vancouver, BC V5Z 4R4, Canada; linda.hoang@bccdc.ca; 12 Alberta Precision Laboratories: Public Health Laboratory (ProvLab), Edmonton, AB T6G 2J2, Canada; linda.chui@albertaprecisionlabs.ca; 13 Department of Laboratory Medicine and Pathology, University of Alberta, Edmonton, AB T6G 2B7, Canada; 14 Roy Romanow Provincial Laboratory, Regina, SK S4S 5W6, Canada; jessica.minion@rqhealth.ca; 15 Newfoundland and Labrador Public Health and Microbiology Laboratory, St. John’s, NL A1A 3Z9, Canada; george.zahariadis@easternhealth.ca

**Keywords:** antimicrobial resistance, whole-genome sequencing, molecular epidemiology, AMR prediction, *Salmonella*

## Abstract

Whole genome sequencing (WGS) of *Salmonella* supports both molecular typing and detection of antimicrobial resistance (AMR). Here, we evaluated the correlation between phenotypic antimicrobial susceptibility testing (AST) and in silico prediction of AMR from WGS in *Salmonella enterica* (*n* = 1321) isolated from human infections in Canada. Phenotypic AMR results from broth microdilution testing were used as the gold standard. To facilitate high-throughput prediction of AMR from genome assemblies, we created a tool called Staramr, which incorporates the ResFinder and PointFinder databases and a custom gene-drug key for antibiogram prediction. Overall, there was 99% concordance between phenotypic and genotypic detection of categorical resistance for 14 antimicrobials in 1321 isolates (18,305 of 18,494 results in agreement). We observed an average sensitivity of 91.2% (range 80.5–100%), a specificity of 99.7% (98.6–100%), a positive predictive value of 95.4% (68.2–100%), and a negative predictive value of 99.1% (95.6–100%). The positive predictive value of gentamicin was 68%, due to seven isolates that carried *aac(3)-IVa*, which conferred MICs just below the breakpoint of resistance. Genetic mechanisms of resistance in these 1321 isolates included 64 unique acquired alleles and mutations in three chromosomal genes. In general, in silico prediction of AMR in *Salmonella* was reliable compared to the gold standard of broth microdilution. WGS can provide higher-resolution data on the epidemiology of resistance mechanisms and the emergence of new resistance alleles.

## 1. Introduction

*Salmonella* spp. are a major cause of foodborne illness that can produce symptoms ranging from mild gastrointestinal illness to more severe invasive infections such as bacteremia. Invasive infections with nontyphoidal *Salmonella* accounted for an estimated 535,000 human cases and 59,100 deaths in 2017 globally [1,2]. While most cases are self-limiting, antimicrobials may be prescribed for invasive infections and for serious gastrointestinal infections in young infants, the elderly, and immunocompromised individuals [3]. Recommended antimicrobial treatments include ceftriaxone, ciprofloxacin, trimethoprim/sulfamethoxazole, or amoxicillin [3].

Antimicrobial resistance (AMR) in *Salmonella*, including multidrug resistance, is increasing [4,5,6,7,8,9]. Surveillance of AMR can help inform treatment guidelines and policies on antimicrobial stewardship in human and veterinary medicine. Traditional antimicrobial susceptibility testing (AST) is performed with phenotypic methods, including broth microdilution, disc diffusion, and Etest strips. Advances in the speed and cost of whole-genome sequencing (WGS) is transforming microbiology [10]. Globally, countries are transitioning to WGS for epidemiological surveillance, including detection of outbreaks, and detection of AMR in *Salmonella* and other pathogens [10]. Genetic mechanisms of resistance are relatively well characterized in *Salmonella*, which facilitates in silico AMR prediction [11].

There are many databases and tools for AMR detection, such as CARD, AMRFinder, ARG-ANNOT, and ARIBA [12,13,14,15]. Each tool has its strengths and limitations, and different countries have adopted different approaches for genotypic AMR detection [10]. The Center for Genomic Epidemiology (CGE) in Denmark has published databases for acquired resistance genes (ResFinder), chromosomal mutations (PointFinder), and plasmids (PlasmidFinder) [16,17,18]. CGE’s web-based tools to query these databases can help low- and middle-income countries to overcome challenges in the implementation of in silico AMR detection; however, the offline versions of these tools do not currently process samples in batches [19].

Here, we describe a new tool called Staramr, which was created to query the CGE databases in high throughput. Staramr uses a BLAST-based approach to scan bacterial genome contigs for antimicrobial genes and mutations and compiles a summary report of genetic mechanisms and predicted antibiogram based on a gene-drug key developed by the United States Centers for Disease Control (US CDC). We used Staramr to evaluate the reliability of in silico AMR detection from WGS for *Salmonella enterica* isolated from human infections in Canada.

## 2. Methods

Bacterial isolates. The Canadian Integrated Program for Antimicrobial Resistance Surveillance (CIPARS) collects human clinical isolates of *Salmonella* from all ten provincial public health laboratories in Canada. Further details on the methods used by CIPARS are described in the Design and Methods section of the annual report [20]. Our study included all isolates of *Salmonella enterica* collected from January to June 2017 that were tested by both broth microdilution and WGS (*n* = 1321).

Antimicrobial susceptibility testing (AST). AST was carried out by broth microdilution using the Sensititer Automated Microbiology System (Trek Diagnostic Systems Ltd., Westlake, OH, USA). We used clinical breakpoints established by the Clinical Laboratory Standards Institute M100:ED27 for all drugs except streptomycin, where an epidemiological cut-off of 64 mg/L was used [21]. Fourteen antimicrobials on the CMV4AGNF panel were tested, including amoxicillin/clavulanic acid (AMC), ampicillin (AMP), azithromycin (AZM), chloramphenicol (CHL), ciprofloxacin (CIP), ceftriaxone (CRO), cefoxitin (FOX), sulfisoxazole (FIS), gentamicin (GEN), meropenem (MEM), nalidixic acid (NAL), streptomycin (STR), sulfisoxazole/trimethoprim (SXT), and tetracycline (TET). This panel is also used by the National Antimicrobial Resistance Monitoring System (NARMS) [22]. CIPARS routinely carries out susceptibility testing on eleven *Salmonella* serotypes (4,[5],12,i:-, Dublin, Enteritidis, Heidelberg, Infantis, Kentucky, Newport, Paratyphi A, Paratyphi B, Typhi, and Typhimurium), which were chosen because they are frequently isolated from human samples or frequently multidrug-resistant. In addition to routine testing of these 11 serotypes, other serotypes were tested by request or for research projects.

Whole-genome sequencing and assembly. PulseNet Canada conducts short-read WGS on all *Salmonella* from human-source infections. DNA extractions were carried out with the Epicentre Complete DNA and RNA Extraction Kit (Illumina Inc, San Diego, CA, USA) or the DNeasy blood and tissue kit (Qiagen, Germantown, MD, USA). Libraries were prepared with the Nextera XT kit and sequencing was carried out on the Miseq platform with the Miseq Reagent v3 600 cycle kit (Illumina Inc, San Diego, CA, USA). Isolates with coverage below 40× and an average Q-score ≤ 30 were re-sequenced. Genomes were assembled within Bionumerics v7.6.3 using spades v3.7.1 [23] with a minimum contig length of 1000. The quality of the assemblies was assessed within BioNumerics v7.6.3; isolates with ≥200 contigs or a genome size outside the range of 4.4 to 6.0 Mb were re-sequenced.

Staramr and genotypic AMR prediction. Determinants of antimicrobial resistance and plasmids were detected with the Public Health Agency of Canada’s Staramr tool, which is available at https://github.com/phac-nml/staramr (date accessed 14 December 2021), as a Python package at https://pypi.org/project/staramr/ (date accessed 14 December 2021), as a bioconda package (name: *staramr*), and also as a tool in the Galaxy bioinformatics analysis platform (https://academic.oup.com/nar/article/46/W1/W537/5001157, date accessed 14 December 2021). The current version of staramr (0.7.2) was also deposited to Zenodo (https://doi.org/10.5281/zenodo.5866712). The current version of Staramr, which was written in the Python programming language, incorporates BLAST [24] ResFinder, PointFinder, and PlasmidFinder databases [16,17] as well as the PubMLST databases (https://pubmed.ncbi.nlm.nih.gov/30345391/, date accessed 3 February 2020) using the software mlst (https://github.com/tseemann/mlst, date accessed 3 February 2020). Staramr v0.7.0 or later also applies quality metrics for the assembly, including: max number of contigs: 1000; min contig length: 300; min N50 length: 10,000; and genome size range: 4 Mb-6.5 Mb. For this study, Staramr v0.2.1 was used with ResFinder database version e8f1eb2585cd9610c4034a54ce7fc4f93aa95535 (July 2018) and PointFinder database version 8706a6363bb29e47e0e398c53043b037c24b99a7 (July 2018). The parameters used for the Staramr query were: percent identity threshold for BLAST: 98, percent length overlap of BLAST hit for ResFinder database: 52, percent length overlap of BLAST hit for PointFinder database: 95.

Sequence accession. PulseNet Canada deposits sequence reads of *Salmonella enterica* to the National Center for Biotechnology Information in Bioproject PRJNA543337.

## 3. Results

Staramr tool for genotypic AMR prediction. Staramr can be used to (1) detect resistance genes and point mutations in assembled contigs, (2) apply a gene-drug key to produce a predicted antibiogram, and (3) provide assembly statistics, plasmids and the MLST type of the genome. The program utilizes CGE’s ResFinder, PointFinder, and PlasmidFinder databases as well as PubMLST databases. Staramr (* amr) was named after the asterisk (star) character, which is often used as a wildcard when searches are performed within software. The gene *aac(6′)-Iaa* was frequently detected but did not confer resistance to any antimicrobial; therefore, the script was revised to reject this gene at the antibiogram prediction step. This gene is reported to be cryptic [25]. For PointFinder, the minimum length was set to 95% to ensure that the correct gene was captured. For ResFinder, a truncated but functional *sul1* was sometimes detected in *S*. Typhimurium; the threshold of minimum length of the BLAST hit was thus lowered to 52% (from the default of 60%) when querying *Salmonella*, which improved overall sensitivity but did not affect specificity. The nucleotide ID threshold for ResFinder was set to 98% ID, as recommended by Zankari et al. [16].

Comparison of phenotypic and genotypic AMR detection. Genotypic AMR prediction was conducted with Staramr, and MICs were produced by broth microdilution testing. We compared phenotypic and genotypic resistance for 14 antimicrobials belonging to ten classes. Staramr predicts categorical resistance (resistant or non-resistant) for all drugs except for ciprofloxacin, which was categorized as either intermediate/resistant or susceptible. Of 1321 isolates, 529 displayed resistance to one or more antimicrobials for a total of 1572 instances of resistance in the dataset.

There was an overall correlation of 99.0% between phenotypic and genotypic detection of categorical resistance for 14 drugs against 1321 isolates (18,305 of 18,494 results in agreement). Using broth microdilution results as the gold standard, genotypic AMR prediction displayed average sensitivity of 91.2% (range 80.5–100%), specificity of 99.7% (98.6–100%), positive predictive value (PPV) of 95.4% (68.2–100%), and negative predictive value (NPV) of 99.1% (95.6–100%) (Table 1).

The sensitivity of detection, which is the ability of the genotypic test to detect antimicrobial resistance (true positive rate), was >90% for 10 antimicrobials: ampicillin, chloramphenicol, ciprofloxacin, ceftriaxone, sulfisoxazole, gentamicin, meropenem, streptomycin, sulfisoxazole/trimethoprim, and tetracycline. The antimicrobials with sensitivity <90% were amoxicillin/clavulanic acid, azithromycin, cefoxitin, and nalidixic acid. For amoxicillin/clavulanic acid, the sensitivity was 87.8% due to 5/41 phenotypically resistant isolates being missed by the genotypic method (three of the five missed isolates contained *bla*_CARB-2_). In our dataset, 41/49 (84%) of isolates containing *bla*_CARB-2_ had amoxicillin/clavulanic acid MIC that was one two-fold dilution below the resistance breakpoint (intermediate category), three were resistant and five were susceptible. Thus, *bla*_CARB-2_ appears to confer reduced susceptibility to amoxicillin/clavulanic that is usually just below the resistance break point. Azithromycin detection had a sensitivity of 83.3% due to 2/10 azithromycin resistant isolates being missed by genotypic detection while cefoxitin displayed a sensitivity of 80.5% due to 8/41 resistant isolates being missed. These exceptions may be due to porin mutations or plasmid loss. For nalidixic acid, 49/251 nalidixic acid resistant isolates were missed by genotypic detection resulting in a sensitivity of 80.5%. Of these 49 false negatives, 32 (65%) contained the quinolone resistance gene *qnrB19*, which Staramr currently predicts as conferring ciprofloxacin intermediate/resistance but not nalidixic acid-resistance. In our dataset, of 35 isolates containing *qnrB19*, 32 isolates were nalidixic acid-resistant, two had MIC of one two-fold dilution below the resistance breakpoint and one was susceptible. If the interpretation of *qnrB19* was changed from only “CIP-I/R” to both “CIP-I/R, and NAL-R”, the sensitivity of detection of nalidixic acid would increase from 80.5% to 93.2%.

The specificity of the genotypic detection of AMR, which is the ability of the genotypic test to detect susceptbility (true negative rate), was high for all drugs, ranging from 98.6% for streptomycin to 100% for azithromycin, meropenem, sufisoxazole, sulfamethoxazole/trimethoprim, and tetracycline.

The PPV is the probability that an isolate that is predicted to be resistant by the genotypic test is actually resistant. This metric differs from sensitivity because it takes into account the prevalence of resistance in a population. The PPVs were >90% for all drugs except gentamicin and cefoxitin. For gentamicin, the PPV of 68.2% was due to 7 genotypic false positive isolates out of 1305 phenotypically susceptible isolates. All seven false positives carried an *aac(3)-IVa* gene and were predicted to be gentamicin resistant but had MICs of one or two two-fold dilutions below the resistance breakpoint. Only two isolates carrying *aac(3)-IVa* were phenotypically resistant. Thus, *aac(3)-IVa* appears to confer MICs close to the CLSI breakpoint for gentamicin. Cefoxitin had a PPV of 89.2% due to 4 genotypic false positives out of 1281 phenotypically susceptible isolates. Three of the four phenotypically susceptible isolates carried *bla*_CMY-2_ and had MICs that were one two-fold dilution below the resistance breakpoint while one isolate carried *bla*_CMY-4_. Thus, both antimicrobials with lower PPVs were mainly due to genes that conferred MICs just below the CLSI resistance breakpoint.

The negative predictive value is the probability that an isolate that is predicted to be susceptible by the genotypic test is actually susceptible. This metric differs from specificity because it takes into account the prevalence of resistance in a population. The negative predictive values for genotypic AMR detection ranged from 95.6% for nalidixic acid to >98% for the other 13 drugs.

Isolates showing discrepancies to ≥2 drugs were retested by broth microdilution, but categorical results did not change significantly (data not shown). Resistance determinants were also detected for antimicrobials that were not routinely tested during the study period such as fosfomycin (*n* = 156), kanamycin (*n* = 140), hygromycin (*n* = 9), lincomycin (*n* = 1), rifampin (*n* = 1) and colistin (*n* = 1). Colistin has since been added to the broth microdilution antimicrobial panel that is used for routine testing by NARMS and CIPARS due to renewed interest in this drug as a last resort treatment for highly drug-resistant organisms.

Mechanisms of resistance. In total, we detected 64 alleles of acquired genes and mutations in three chromosomal genes (Table 2). For beta-lactams (ampicillin, amoxicillin/clavulanic acid, cefoxitin, ceftriaxone, or meropenem) 13 unique beta-lactamase genes were identified, with most conferring resistance to ampicillin only. No determinants for meropenem resistance were detected, consistent with MIC testing. The most common ampicillin resistance genes were *bla*_TEM-1B_ (*n* = 90) and *bla*_CARB-2_ (*n* = 49) (Table 2). We detected AmpC-type beta-lactamases encoded by *bla*_CMY-2_ (*n* = 33), *bla*_CMY-44_ (*n* = 2), *bla*_CMY-4_ (*n* = 1), and *bla*_CMY-54_ (*n* = 1), which confer resistance to all of the beta-lactams tested except meropenem. We also detected the extended spectrum beta-lactamase (ESBL) alleles *bla*_CTX-M-65_ (*n* = 8), *bla*_CTX-M-55_ (*n* = 3), and *bla*_CTX-M-9_ (*n* = 1). For folate pathway inhibitors, resistance was mediated by alternative targets that are insensitive to drugs. Genes *sul1* (*n* = 95) and *sul2* (*n* = 103) accounted for 96% (198/206) of sulfisoxazole resistance, while *dfrA1* (*n* = 13), *dfrA7* (*n* = 11), *dfrA12* (*n* = 8), and *dfrA14* (*n* = 8) accounted for 85% (40/47) of sulfamethoxazole/trimethoprim resistance. For quinolones, ciprofloxacin non-susceptibility (intermediate and resistant categories) was conferred by plasmid-mediated quinolone resistance (PMQR) determinants, which protect the DNA-enzyme complex from inhibition. We detected the PMQR genes *qnrB19* (*n* = 37), *qnrS1* (*n* = 8), *qnrA1* (*n* = 5), *qnrB6* (*n* = 1)) and *aac(6′)-Ib*-cr (*n* = 1). Mutations in the quinolone resistance determining regions (QRDR) of the targets *gyrA*, *gyrB* and *parC* were predicted to confer both nalidixic acid resistance and ciprofloxacin non-susceptibility. QRDR mutations in *gyrA* were the most common and included S83F (*n* = 100), D87N (*n* = 58), D87Y (*n* = 28), S83Y (*n* = 21) and D87G (*n* = 11) variants. The *gyrB* variants E466D (*n* = 13) and (S464Y) (*n* = 2), and the *parC* variants S80I (*n* = 17) and E84G (*n* = 1) were also detected.

Several classes of antimicrobials that act on the ribosome were tested. For aminoglycosides (gentamicin and streptomycin), 15 unique genes were identified encoding aminoglycoside-modifying enzymes belonging to all three classes (acetyltransferases, nucleotidyltransferases, and phosphotransferases). Genes *aac(3)-IVa* (*n* = 9) and *aac(3)-VIa* (*n* = 8), accounted for 74% (17/23) of gentamicin resistance, while *aph(3″)-Ib* (*n* = 91), *aadA2* (*n* = 64), and *ant(3″)-Ia* (*n* = 19) accounted for 90% (174/193) of streptomycin resistance. For the macrolide category, resistance to azithromycin was conferred by the antibiotic inactivation genes *mph(A)* (*n* = 9) and *erm(B)* (*n* = 1), while in the phenicol category, resistance to chloramphenicol was conferred by the *floR* (*n* = 79) transporter and *catA1* (*n* = 11) inactivation enzyme in 89% (90/101) of isolates. Tetracycline resistance was conferred by genes encoding efflux proteins (*tet(A*) (*n* = 68), *tet(B)* (*n* = 55), and *tet(G)* (*n* = 44)), and the ribosomal protection enzyme *tet(M)* (*n* = 4).

## 4. Discussion

We developed and evaluated the use of an AMR prediction tool called Staramr and observed a strong correlation between phenotypic and genotypic detection of AMR in *Salmonella*. One advantage of Staramr over similar programs is that it evaluates quality metrics of the input data. Staramr, which uses genome assemblies as input, is not computationally intensive, so that it can be run on a local computer instead of a high-performance cluster. We recommend the use of a quick genome assembler such as Shovill/SPAdes (Shovill: https://github.com/tseemann/shovill, date accessed 18 January 2022; SPAdes: https://currentprotocols.onlinelibrary.wiley.com/doi/abs/10.1002/cpbi.102, date accessed 18 January 2022) [23]. Future work will include adding point mutations for additional bacterial species from the PointFinder database. Further, antibiogram predictions are an experimental feature which is continually being improved. The CARD database is not as yet validated in terms of which genes/mutations confer MICs above a clinical resistance breakpoint; however, CARD was useful for further analysis of discrepant results.

Genotypic AMR prediction is currently used for surveillance in some countries, but further validation of its reliability is needed before this test is approved by regulatory agencies for clinical use. Genotypic results, like phenotypic results, would need to be subjected to clinical interpretation of which antimicrobials are appropriate for treatment. The overall concordance between genotypic and phenotypic AMR detection was 99% in *Salmonella* isolates in Canada. Similar studies in *Salmonella* from Denmark and the United States have found concordances of 98.8% (slightly lower for quinolone) and 99% (slightly lower for aminoglycosides and beta-lactams), respectively [19,26]. The NCBI AMRFinder tool found a concordance of 98% for *Salmonella* [13].

The positive and negative predictive values of genotypic detection of AMR were >95% for all antimicrobials except gentamicin and cefoxitin. For these two drugs, a few false positive results were obtained from isolates with known resistance genes that conferred MICs just below the current CLSI breakpoints for resistance. A study by McDermott et al. found that the greatest number of discrepancies occurred for aminoglycosides and beta-lactams, notably streptomycin and cefoxitin [26]. Similar to our findings, cefoxitin false positives in their study carried resistance genes that conferred MICs just below the resistance breakpoint. If an infection was caused by an isolate known to carry a resistance gene, that drug may not be the first choice for treatment despite the MIC being slightly below the breakpoint in in vitro testing. The European Committee for Antimicrobial Susceptibility Testing (EUCAST) also provides clinical breakpoints for *Salmonella*, and, in some cases, the breakpoints are different from CLSI [27]. While phenotypic testing is considered to be the gold standard, there are several caveats for this method. Variability in phenotypic results occur due to multiple factors such as amount of inoculum used, and bacterial growth conditions. Further, the conditions used in vitro do not perfectly mimic the site of infection.

The gene-drug key is provided with support from the US CDC and is continually being improved. There were 141 false negatives whereby isolates that were phenotypically resistant were not predicted to be resistant in the genotypic test. One fifth of these discrepancies might be eliminated by modifying the gene-drug key to interpret *qnrB19* as both CIP-I/R and NAL-R instead of CIP-I/R only. Optimization of the key may be influenced by strain epidemiology in different geographic locations. In the United States, *qnrB19* does not reliably confer nalidixic acid resistance, perhaps due to different serotype or strain epidemiology in Canada and the United States. In some cases, such as *bla*_CARB-2_ which usually conferred MIC just below the resistance breakpoint, the current interpretation is appropriate for the CLSI breakpoint, but variation in phenotypic testing or strain dependence can cause occasional discrepancies. False negatives may also be caused when strains carry determinants of resistance that are currently undiscovered or fragmented in the assembly; however, large-scale WGS along with continued phenotypic testing of a subset of isolates offers opportunities to discover new mechanisms of resistance to strengthen the databases.

Mechanisms of resistance detected in our dataset included 64 unique acquired alleles and mutations in three genes (*gyrA*, *gyrB*, and *parC*) conferring resistance to all antimicrobials that were phenotypically tested except for meropenem. These resistance mechanisms were mostly similar to those reported in a similar study from the United States, which detected 65 unique mechanisms [26]. Some of the differences in the two studies may be due to the fact that Canadian isolates were all from human sources, whereas the majority of isolates in the US study were from food/animals.

For ciprofloxacin, the intermediate and resistant categories were combined, and the gene-drug key interprets the presence of any determinant as CIP-I/R. In general, the presence of a single fluoroquinolone resistance determinant such as a *gyrA* mutation or a *qnr* allele conferred an MIC in the intermediate range, while the presence of multiple determinants conferred outright resistance. There was a lot of variability in this general observation; the intermediate and resistant categories were therefore combined. Studies using machine learning or deep learning approaches with large datasets are being conducted to produce algorithms for predicting antimicrobial MICs for *Salmonella enterica*, *Neisseria gonorrhoeae*, *Klebsiella pneumoniae*, and other pathogens [28,29,30].

One limitation of genotypic-based detection of AMR is the possibility of missing new resistance genes; thus, CIPARS continues to test 10% of human-source *Salmonella* using broth microdilution for continual validation and detection of emerging resistance. On the other hand, WGS will facilitate the detection of new variants of known resistance genes. WGS also allows monitoring of the molecular epidemiology of resistance mechanisms in different reservoirs to enhance One Health studies of AMR. Genomic databases can be scanned retroactively when new mechanisms of resistance are discovered; for example, many institutions conducted retrospective analyses after the discovery of the first mobile colistin resistance (*mcr-1*) gene. Genotypic AMR detection also allows monitoring of trends in specific combinations of genes that produce different multidrug resistance patterns, as well as monitoring the genetic context of resistance genes, and potential for horizontal gene transfer.

In summary, in silico prediction of AMR from WGS for *Salmonella* from humans in Canada was reliable. This method can yield a wealth of additional data that is not routinely generated from phenotypic testing, such as the genetic context of resistance and surveillance of the molecular epidemiology of resistance determinants.

## Figures and Tables

**Table 1:** Comparison of phenotypic and in silico AMR prediction in *Salmonella enterica* (*n* = 1321) for 14 antimicrobials.

	Phenotype Resistant ^a^		Phenotype Susceptible ^a^						
Class and Antimicrobial ^c^	Genotype Resistant	Genotype Susceptible	Genotype Resistant	Genotype Susceptible	Concordance (%)	Sensitivity (%) ^b^	Specificity (%) ^b^	PPV (%) ^b^	NPV (%) ^b^
Aminoglycoside									
GEN	15	1	7	1298	99.4	93.8	99.5	68.2	99.9
STR	162	16	16	1127	97.6	91.0	98.6	91.0	98.6
Beta-lactam/beta-lactam inhibitor									
AMC	36	5	1	1279	99.5	87.8	99.9	97.3	99.6
Carbapenem									
MEM	0	0	0	1321	100.0	100.0	100.0	100.0	100.0
Cephem									
FOX	33	8	4	1277	99.2	80.5	99.7	89.2	99.5
CRO	48	3	1	1269	99.7	94.1	99.9	98.0	99.8
Folate pathway inhibitors									
FIS	179	9	0	1133	99.3	95.2	100.0	100.0	99.2
SXT	47	5	0	1269	99.6	90.4	100.0	100.0	99.6
Macrolide									
AZM	10	2	0	1309	99.8	83.3	100.0	100.0	99.8
Penicillin									
AMP	182	9	2	1128	99.2	95.3	99.8	98.9	99.2
Phenicol									
CHL	95	5	1	1220	99.5	95.0	99.9	99.0	99.6
Quinolones									
CIP I/R	259	20	2	1040	98.3	92.8	99.8	99.2	98.1
NAL	202	49	13	1057	95.3	80.5	98.8	94.0	95.6
Tetracycline									
TET	162	10	0	1149	99.2	94.2	100.0	100.0	99.1
Total/Average	1431	141	47	16,876	99.0	91.2	99.7	95.4	99.1

^a^ Resistant and susceptible phenotypes were determined by broth microdilution testing and interpreted according to CLSI guidelines; for streptomycin a breakpoint of 64 mg/L was used and for ciprofloxacin the intermediate and resistant categories were combined. ^b^ Sensitivity, specificity, positive predictive value (PPV), and negative predictive value (NPV) were calculated for the genotypic test using broth microdilution as the gold standard ^c^ Antimicrobials tested were amoxicillin/clavulanic acid (AMC), ampicillin (AMP), azithromycin (AZM), chloramphenicol (CHL), ciprofloxacin (CIP), ceftriaxone (CRO), cefoxitin (FOX), sulfisoxazole (FIS), gentamicin (GEN), meropenem (MEM), nalidixic acid (NAL), streptomycin (STR), sulfisoxazole/trimethoprim (SXT), and tetracycline (TET).

**Table 2:** Genetic mechanisms of resistance detected in *Salmonella enterica* (*n* = 1321).

Predicted Phenotype	Genetic Resistance Determinants
Aminoglycosides	
gentamicin (*n* = 23)	*aac(3)-IVa* (*n* = 9), *aac(3)-VIa* (*n* = 8), *aac(3)-Id* (*n* = 3), *aac(3)-IId* (*n* = 2), *aac(6′)-Ib-cr* (*n* = 1)
gentamicin, kanamycin (*n* = 1)	*ant(2″)-Ia* (*n* = 1)
streptomycin (*n* = 193)	*aph(3″)-Ib* (*n* = 91), *aadA2* (*n* = 64), *ant(3″)-Ia* (*n* = 19), *aadA1* (*n* = 9), *strA* (*n* = 4), *aadA7* (*n* = 3), *aadA16* (*n* = 1), *aadA22* (*n* = 1), *aph(6)-Ic* (*n* = 1)
Beta-lactams	
ampicillin (*n* = 146)	*bla*_TEM-1B_ (*n* = 90), *bla*_CARB-2_ (*n* = 49), *bla*_TEM-206_ (*n* = 3), *bla*_TEM-1A_ (*n* = 2), *bla*_TEM-1C_ (*n* = 1), *bla*_TEM-90_ (*n* = 1)
ampicillin, amoxicillin/clavulanic acid, cefoxitin, ceftriaxone (*n* = 37)	*bla*_CMY-2_ (*n* = 33), *bla*_CMY-44_ (*n* = 2), *bla*_CMY-4_ (*n* = 1), *bla*_CMY-54_ (*n* = 1)
ampicillin, ceftriaxone (*n* = 12)	*bla*_CTX-M-65_ (*n* = 8), *bla*_CTX-M-55_ (*n* = 3), *bla*_CTX-M-9_ (*n* = 1)
Folate Pathway Inhibitors	
sulfisoxazole (*n* = 206)	*sul2* (*n* = 102), *sul1* (*n* = 95), *sul3* (*n* = 9)
trimethoprim (*n* = 47)	*dfrA1* (*n* = 13), *dfrA7* (*n* = 11), *dfrA12* (*n* = 8), *dfrA14* (*n* = 8), *dfrA15* (*n* = 3), *dfrA5* (*n* = 3), *dfrA27* (*n* = 1)
Macrolides	
erythromycin, azithromycin (*n* = 10)	*mph(A)* (*n* = 9), *erm(B)* (*n* = 1)
Phenicol	
chloramphenicol (*n* = 101)	*floR* (*n* = 79), *catA1* (*n* = 11), *cmlA1* (*n* = 6), *catA2* (*n* = 3), *oqxA* (*n* = 1), *oqxB* (*n* = 1)
Quinolones	
ciprofloxacin I/R (*n* = 52)	*qnrB19* (*n* = 37), *qnrS1* (*n* = 8), *qnrA1* (*n* = 5), *aac(6′)-Ib-cr* (*n* = 1), *qnrB6* (*n* = 1)
ciprofloxacin I/R, nalidixic acid (*n* = 251)	*gyrA* (S83F) (*n* = 100), *gyrA* (D87N) (*n* = 58), *gyrA* (D87Y) (*n* = 28), *gyrA* (S83Y) (*n* = 21), *parC* (S80I) (*n* = 17), *gyrB* (E466D) (*n* = 13), *gyrA* (D87G) (*n* = 11), *gyrB* (S464Y) (*n* = 2), *parC* (E84G) (*n* = 1)
Tetracycline	
tetracycline (*n* = 171)	*tet(A)* (*n* = 68), *tet(B)* (*n* = 55), *tet(G)* (*n* = 44), *tet(M)* (*n* = 4)
Not tested phenotypically	
fosfomycin (*n* = 156)	*fosA7* (*n* = 150), *fosA3* (*n* = 6)
kanamycin (*n* = 140)	*aph(6)-Id* (*n* = 96), *aph(3′)-Ia* (*n* = 42), *aph(3′)-IIa* (*n* = 2)
hygromicin (*n* = 9)	*aph(4)-Ia* (*n* = 9)
lincomycin (*n* = 1)	*lnu(G)* (*n* = 1)
rifampicin (*n* = 1)	*ARR-3* (*n* = 1)
erythromycin (*n* = 1)	*mph(B)* (*n* = 1)
colistin (*n* = 1)	*mcr-3* (*n* = 1)

## Data Availability

PulseNet Canada deposits sequence reads of *Salmonella enterica* to the National Center for Biotechnology Information in Bioproject PRJNA543337.
